# Recent Progress in Greenland Ice Sheet Modelling

**DOI:** 10.1007/s40641-017-0073-y

**Published:** 2017-11-13

**Authors:** Heiko Goelzer, Alexander Robinson, Helene Seroussi, Roderik S.W. van de Wal

**Affiliations:** 1 Institute for Marine and Atmospheric Research Utrecht (IMAU), Utrecht University, Princetonplein 5, 3584 CC Utrecht, The Netherlands; 2 Laboratoire de Glaciologie, Université Libre de Bruxelles, Brussels, Belgium; 3 Faculty of Geology and Geoenvironment, University of Athens, 15784 Athens, Greece; 4 Universidad Complutense de Madrid, 28040 Madrid, Spain; 5 Instituto de Geociencias, UCM-CSIC, 28040 Madrid, Spain; 6 Jet Propulsion Laboratory, California Institute of Technology, Pasadena, CA USA

**Keywords:** Greenland ice sheet, Numerical modelling, Ice dynamics, Ice thermodynamics, Ice sheet–climate interactions, Sea-level rise

## Abstract

**Purpose of Review:**

This paper reviews the recent literature on numerical modelling of the dynamics of the Greenland ice sheet with the goal of providing an overview of advancements and to highlight important directions of future research. In particular, the review is focused on large-scale modelling of the ice sheet, including future projections, model parameterisations, paleo applications and coupling with models of other components of the Earth system.

**Recent Findings:**

Data assimilation techniques have been used to improve the reliability of model simulations of the Greenland ice sheet dynamics, including more accurate initial states, more comprehensive use of remote sensing as well as paleo observations and inclusion of additional physical processes.

**Summary:**

Modellers now leverage the increasing number of high-resolution satellite and air-borne data products to initialise ice sheet models for centennial time-scale simulations, needed for policy relevant sea-level projections. Modelling long-term past and future ice sheet evolution, which requires simplified but adequate representations of the interactions with the other components of the Earth system, has seen a steady improvement. Important developments are underway to include ice sheets in climate models that may lead to routine simulation of the fully coupled Greenland ice sheet–climate system in the coming years.

## Introduction

The Greenland ice sheet (GrIS) is the second largest ice body on Earth and is expected to be a major contributor to future sea-level rise [1]. Potential threshold behaviour in response to a warming Arctic may cause a long-term and irreversible retreat of the ice sheet for warming levels that could be realised within this century [2–4]. Understanding its past, present and future behaviour is therefore of both great scientific interest and societal concern.

**Fig. 1:**
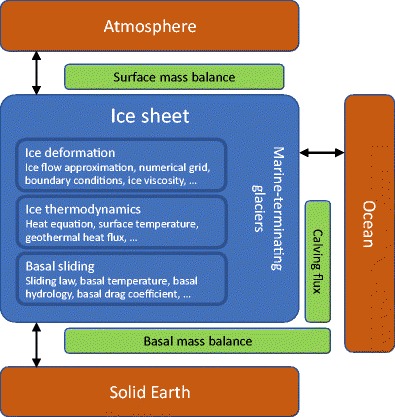
Elements of a typical Greenland ice sheet model (blue) and possible interactions with the other components of the Earth system (orange). Mass exchange processes are shown in green. Changes in ice sheet size, mass and local ice thickness affect the properties of atmosphere, ocean and solid Earth and vice versa. Key processes for the ice sheet model itself are the ice temperature evolution and the flow of the ice caused by deformation and sliding, depending primarily on ice thickness, surface slope and ice temperature

Projecting future changes and studying the past behaviour of the GrIS and its interactions with other components of the Earth system requires numerical ice sheet models built around a physical description of ice sheet flow (Fig. 1). In its present state, the GrIS gains mass by snow accumulation at higher elevations and flows by deformation under its own weight and by sliding over a bed of rock and/or sedimentary material. At its margins, the ice sheet loses mass mainly by surface meltwater runoff, ocean-driven melting and iceberg calving from a large number of marine-terminating outlet glaciers. Its total mass change is determined by the sum of the surface mass balance (SMB), basal mass balance (melting or freezing under the ice sheet) and mass exchange of the marine-terminating outlet glaciers with the ocean, today predominantly occurring in the form of calving of icebergs that eventually melt in ocean waters [5].

The rate of ice deformation is strongly dependent on ice thickness, surface slope and rheological properties of the ice and the underlying substrate, which change considerably with changing temperature, impurity concentration and water content of the ice. Thermodynamic coupling is needed to model the long-term (millennial to multi-millennial) evolution as the ice sheet responds to climate changes and might only be neglected for short-term (decadal to centennial time scale) simulations. Another long-term interaction mechanism of the ice sheet concerns isostatic bedrock adjustment under the varying load of the ice. The millennial to multi-millennial response time scales associated with bedrock changes and thermodynamics have led to the use of spin-up techniques that consist of running ice sheet models with boundary conditions of simulated or reconstructed past climate changes to capture the long-term memory of these processes [4, 6].

However, the present-day configuration of the ice sheet simulated with such long-term reconstructions shows a rather large discrepancy when compared with available satellite and airborne observations [7, 8]. Studies focusing on current mechanisms affecting ice sheet dynamics and short-term evolution of ice sheets also need an accurate representation of the present-day ice sheet configuration and may therefore rely on observations to initialise models: they infer poorly known englacial or subglacial parameters and boundary conditions using data assimilation techniques of observed surface conditions [9, 10].

GrIS numerical models vary largely in terms of the processes they include, the spatial resolution of their underlying grids and the forcings they apply [11] depending on the time periods of interest and the scientific questions they address. This is similar for the level of detail contained in simulated interactions with the other components of the Earth system. Climatic boundary conditions may, e.g., be prescribed, estimated from simple parameterisations or calculated with full complexity climate models, with either one-directional or two-way coupling between ice sheet and climate models.

Acknowledging considerable overlap between them, we discuss three different approaches to GrIS modelling in this paper: (1) The long-term modelling approach with thermodynamic coupling and isostatic bedrock adjustment, requiring spin-up techniques, (2) data assimilation techniques, typically applied to model initialisation for centennial time-scale projections, making extensive use of high-resolution observations of the present-day ice sheet with inversion techniques, (3) fully coupled ice sheet–climate modelling of different levels of complexity to physically represent feedbacks between the ice sheet, atmosphere and ocean. All three modelling approaches, for example, are represented in the GrIS model initialisation experiments (initMIP-Greenland) [12] performed within the context of the Ice Sheet Model Intercomparison Project for CMIP6 (ISMIP6) [13]. It should be noted that many of the issues discussed here could be equally applied to other ice sheet domains, like Antarctica [14].

Distinguishing between the different approaches is somewhat arbitrary but, in many cases, derives from the specific goals of individual research groups. In fact, closer collaboration across these boundaries could further advance the ice sheet modelling community and should be encouraged. None of the modelling approaches can be completely separated from the others. Aside from appreciating the strengths and limitations of the different approaches for specific model applications, the aim of this review paper is therefore also to stimulate possible directions of future research and cross-fertilisation between different groups. It is in this spirit that we have decided to discuss the different research priorities in one review paper.

## Ingredients of Numerical Models for the Greenland Ice Sheet

In this section, we review recent developments concerning specific aspects of large-scale GrIS modelling.

### Discretisation and Grid Representation

Domain discretisation is central to any numerical problem. Models used for long-term simulations nowadays typically apply regular equidistant grids at a horizontal resolution of 5–20 km [15–17], but very high resolutions have been achieved with massively parallel computational efforts [8]. Finite element grids of variable spatial resolution, with the smallest elements sometimes below kilometre-scale resolution, are also applied, mainly for short-term simulation and data assimilation problems [18–21]. In order to capture critical areas while keeping computational resources manageable, some models have integrated a relatively new approach for large-scale ice sheet simulations by including an adaptive mesh that is updated during runtime, following grounding line or ice front migration [22]. This technique has been applied to capture details of outlet glacier behaviour with a local resolution of 500 m, while a base grid of 8 km is used for most of the ice sheet [23].

### Approximations to the Stress Balance Equation

As ice is a viscous incompressible fluid, it is best modelled using full-Stokes equations. Due to the intensive computational resources needed to solve these equations and the thin aspect ratio of ice sheets, several approximations based on asymptotic developments of these equations have been derived [24]. These can be used to reduce computation time substantially, allowing large-scale simulations to be run with limited resources [9, 25]. Interior regions dominated by vertical shearing are captured well with these simple approximations, while fast-flowing outlet glaciers at the ice sheet margins are best calculated using the full-Stokes solution [26, 27]. Several techniques have been used to combine different approximations [28–30].

While a Stokes model without any approximation remains the best description of ice sheet flow and has been successfully applied for the GrIS on a century time scale [31, 32], it is by now appreciated that certain approximations and combinations thereof represent feasible alternatives to describe full ice sheet–shelf systems on large scale [33]. This is, in particular, the case in the light of other large uncertainties introduced, e.g. by the SMB forcing and other boundary conditions that have a larger impact on ice sheet evolution than choices made concerning the stress balance approximations [31]. Recent attempts have been made to simulate the ice sheet by relying on the shallow shelf approximation in combination with extensive data assimilation techniques [20, 34, 35]. However, the potential consequences of using these approximations to simulate ice dynamics for past and future ice sheet evolution are not well known.

### Thermodynamics and Ice Rheology

Ice sheet thermodynamics are an important element of long-term ice sheet modelling, since ice deformation is non-linearly dependent on ice temperature. In addition, large parts of the GrIS are at the pressure melting point, implying an active role for basal sliding as well. In order to compute both cold and temperate ice properties with a single energy-conserving framework, many models now included an enthalpy framework [36] and a rheology that accounts for water fraction in temperate ice [37]. Enthalpy models compute significantly larger basal melt rates and thinner temperate ice thickness than cold ice models [36].

To aid understanding the evolution of the ice sheet interior, thermodynamic modelling has been used to explain the reconstructed temperature recovered from a Greenland deep ice core [38]. The results indicate that the model can only be reconciled with reconstructions when a combination of different processes, including strain heating and cryo-hydrologic warming in deep crevasses, induced by water infiltration and latent heat exchange [39], is invoked.

Anisotropy is another potential source of discrepancies between observed and modelled ice properties, as rheological laws used in most ice sheet models rely on the isotropic Glen’s flow law [40]. Deep ice cores and radar observations suggest more complex ice rheologies [41, 42] that might explain observed large-scale ice folding [43].

An evaluation of the impact of observationally constrained thermal boundary conditions on the modelled thermomechanical state in western Greenland has shown that there is a strong influence of the surface thermal boundary [44].

A study of the geothermal heat flux distribution beneath the GrIS [45] concludes that present-day basal melt rates and their expression at the surface, like the North East Greenland Ice Stream (NEGIS), may be related to anomalies arising from plate tectonic movements dating back tens of millions of years. Finally, the long-term future evolution of the ice sheet thermal state has been schematically simulated with a thermodynamic model [46] to study the possibility of a thermal-viscous collapse, which implies that the ice sheet disintegration is accelerated by warming of the ice. Results indicate that the lower part of the ice column where most of the deformation occurs could be warmed to the pressure-melting point within a few centuries [46].

### Ice Sheet Basal Conditions

The thermal condition at the base of the ice sheet is known to have important consequences for basal meltwater production and hence for the basal friction and sliding velocity [47]. This view is extended with a study on the sliding of temperate basal ice that may contribute significantly to ice flow in some regions [48]. However, even the basal thermal state (whether ice is frozen or thawed) of the GrIS is poorly known, with at least one third of its area being highly uncertain [49]. Aside from thermal conditions, the basal topography exerts an important control on the flow speed of Greenland outlet glaciers, as shown for Humboldt glacier [50]. Like the thermal state, the basal topography is difficult to map. In general, the method of choice for improving basal topography data sets is to apply mass conservation constraints based on data assimilation techniques [51] (see the “Data Assimilation Techniques” section). To ensure that basal topography data sets are applicable for long-term simulations with significant ice-front advance, there is a need to extend them outside of the present-day ice extent into the fjords and onto the continental shelf [52]. In places where the subglacial topography is poorly known, it could possibly be improved using a trough-system algorithm [53].

### Marine-Terminating Glacier Dynamics

As the GrIS mass loss continues to accelerate [54], most dynamic changes are happening in marine-terminating outlet glaciers, caused by evolution of ice-front positions [55]. Comprehensive treatment of calving processes in large-scale models remains an active field of research. Processes governing calving are complex and include ice properties as well as atmospheric, oceanic and sea ice conditions [56, 57]. The numerical implementation of moving ice fronts in ice flow models is also difficult in unstructured meshes as it requires tracking ice-front positions at sub-element scale and a grid resolution of hundreds of meters [58], which is computationally demanding for continental-scale simulations. Recent developments, relying on the level-set method or linear fracture mechanics, now allow to simulate the evolution of outlet glaciers terminus position [58, 59]. In Greenland, calving is best reproduced using tensile stresses [60], and regional model studies of Jakobshavn Isbræ reproduce the observed acceleration of the glacier over the last three decades when prescribing the ice front evolution [61, 62].

In addition, the interaction of marine-terminating glaciers with ocean water is recognised as an important forcing mechanism, as oceanic conditions impact both submarine melting and calving. However, modelling such interaction remains a challenging problem, mainly due to the large number of poorly mapped fjord geometries, properties of ocean circulation in the fjords and limited observations of melting along ice fronts. In the absence of detailed physically based methods to estimate interactions between outlet glaciers and the ocean explicitly, parameterisations of the bulk effect of the ice-ocean interaction in Greenland fjords have been developed [16, 63]. They represent a simple approach to match model simulations of recent outlet glacier changes to observations and allow projecting the response to both atmospheric and oceanic processes and to estimate the relative importance of the two [16]. The method is a time-dependent extension of an earlier technique that improved the representation of outlet glaciers in a large-scale model with ad hoc adjustment of the sliding relation [64]. More recently, a new empirical discharge parameterisation has been developed [15], with the main goal of improving the simulated extent and margin positions of the ice sheet in a freely evolving model run. Another approach was taken by generalising results from explicit simulations of a limited number of outlet glaciers by a 1D flowline modelling approach [65] and extending the results to the entire ice sheet [66].

## Ice Sheet Model Initialisation and Validation

An important driver for recent development in the field of GrIS modelling is improving physically based projections of the ice sheet’s contribution to future sea-level change. Many ice sheet modelling studies build further on the legacy of the SeaRISE [7, 11] and ice2sea [32, 66–69] projects that provided a set of consistent experiments across models and improved estimates of the ice sheet contribution to sea-level rise for the fifth assessment report of the IPCC [1]. One major conclusion from these projects was the significant impact on centennial ice sheet simulations of differences in model representation of the present-day ice sheets, largely resulting from the initialisation process. Initial ice sheet geometry, velocity structure, basal conditions, englacial properties and imbalance are all critical parameters that should ideally match observations as closely as possible to correctly represent the dynamic state of the ice sheet with confidence. We summarise below the main approaches currently adopted for model initialisation and detail the observations used to validate these models.

### Forward Modelling Approaches

Traditionally, long-term modelling approaches including thermodynamic coupling and isostatic bedrock adjustment were used to provide reconstructions of the present-day ice sheet. Using such a spin-up method, an attempt has been recently made to re-examine the SeaRISE experiments (that were difficult to interpret) with one specific model that is systematically perturbed [17]. Results of this study show that differences in the treatment of the SMB calculations and ice sheet initialisation should have the largest impact on the spread of the ice volume changes in the SeaRISE projections. Previous studies comparing initialisation techniques [70, 71] reached similar conclusions concerning the large impact of different methods. These studies [17, 70, 71] all operate with ice sheet models using a forward modelling approach during initialisation, where ice thickness during (part of) the initialisation process is either free to evolve, fixed to the observed or nudged to the observed.

A relatively good match with the observed velocity structure [72, 73] and ice fluxes at the marine margin [74] with such forward model simulations can be achieved without spatially explicit tuning, and results are improved with increasing grid resolution [16]. However, velocity features that are largely caused by local variations in basal conditions that are not related to geometric features like the NEGIS have not been resolved in this type of model based on a freely evolving spin-up. Furthermore, the observed geometry is generally poorly matched without some form of data assimilation, particularly at the margins, where the simulated ice front can be located tens of kilometres away from its present location. This mismatch makes it challenging to properly capture the hypsometry and consequently the mass balance height feedback in future projections. In addition, external SMB products are typically limited to the observed extent, further complicating the simulations if changes in ice-sheet area play a role.

In principle, an almost perfect match with the observed geometry can be achieved with nudging techniques [8, 70, 71] that apply spatially explicit ice flux corrections in places where the modelled ice sheet does not match observations. However, this method has the great disadvantage that the model is not mass conserving, internally inconsistent and exhibits large and rapid drift when the correction is removed [71]. In addition, it falsifies the dynamic response in a forward experiment, as has been shown earlier [66]: the mass fluxes implied by the correction are held constant in time and therefore do not interact with changing ice sheet geometry and boundary conditions. The decisive question when applying this type of method is then how large the correction is in comparison, e.g. with the prescribed SMB or with the mass fluxes at marine-terminating outlet glaciers. Nevertheless, such models may be used successfully for diagnostic velocity solutions, especially at very high spatial resolution [8].

A different approach to improve the match with the observed velocity and geometry consists of allowing for spatially explicit adjustment of model parameters [75], here still in the framework of the forward modelling approach. While regionally optimising the parameters of the positive-degree-day SMB model may largely compensate for deficiencies in the simulated present-day SMB, the improved match with observed velocities in this model [75] is mainly achieved by iteratively updating the applied basal drag coefficients using an inverse method [76]. These methods rely on the concept that the system is fully understood except for one key parameter, in this case the basal coefficient. This parameter is then spatially varied during the initialisation process and arbitrarily held constant in time for projections of future ice sheet evolution. These models therefore capture the overall dynamics of the present-day state, which is not the case with ice sheet models using traditional spin-up techniques.

### Data Assimilation Techniques

A second category of techniques relies entirely on observations of the ice sheet. Using observations of ice sheet geometry and assuming the ice rheology (and therefore ice temperature, englacial properties, crystal orientation and impurities) is known, basal friction can be treated as the only unknown parameter. In that case, ice sheet initialisation becomes a time-independent data assimilation problem, with the goal of finding optimal model parameters that minimise the misfit with observations, usually surface velocities [10, 29, 32, 77–79]. This approach provides modelled velocities close to observations but causes large unphysical thinning and thickening rates and model drift in forward simulations [29], not as a response to model forcing but as an unphysical response to inconsistencies between data sets. Inconsistencies between the high spatial resolution velocity maps and the low-resolution ice thickness maps, where thickness between observations acquired along flight lines are linearly interpolated, are the main cause of flux divergence anomalies. A model may be relaxed for decades to centuries in order to produce a self-consistent initial state. However, relaxed models may deviate from the target initial conditions and are impacted by the choice of SMB forcing [19, 20, 23, 32, 34].

A new technique to improve basal topography between observations acquired along flight lines was designed, based on the continuity equation and taking advantage of the global coverage in surface velocities [80]. In the mass conservation method [80], ice thickness is first estimated by combining sparse observations of ice thickness with observed surface velocities as well as thinning rates and modelled SMB under the constraint of mass conservation and assuming ice flow is governed by sliding. This multi-parameter optimisation adjusts depth-averaged velocity and apparent mass balance within the observational error in order to minimise the difference between observed and modelled velocities along flight tracks [80]. The method was applied to most outlet glaciers around Greenland [51], allowing significant improvements in the modelling of the velocity fields [8].

The optimisation of the basal topography can also be directly integrated into the data assimilation procedure, either by applying the shallow shelf approximation [35] or a higher-order, non-depth-integrated ice flow model [81]. In the latter, assuming thermomechanical equilibrium with a specified, equilibrium climate forcing, this adjoint-based method combines the misfit with observed surface velocities and ice thickness change in the cost function to be minimised. Combining these different optimisations in a single assimilation procedure allows self-consistency of initial conditions no matter the choice of observations, while models relying on bedrock topography derived with mass conservation should work with observations similar to those used in the optimisation scheme in order to best match observations. However, the combined optimisation typically relies on flux divergence minimisation to adjust bedrock topography [81], which thus does not take advantage of existing thickness measurements and therefore might create unrealistic topographic features.

Instead of only relying on a modelled ice temperature distribution to define the viscosity, one can complement inversion of the basal drag coefficient with an additional optimisation of the stiffness factor [23]. This factor is a parameter in the calculation of the effective viscosity that determines the rheological properties of the ice, which may be dependent on anisotropy, impurities and ice fabric. In this approach, poorly constrained basal and englacial parameters are optimised at the same time, again minimising mismatch of modelled and observed surface velocities. For ice shelves, where there is no unknown basal friction, inversion of ice shelf rheology has been performed to accurately reproduce their flow [29].

As time series of surface elevation and velocity observations become available, a recent development in centennial scale ice sheet modelling concerns techniques that leverage *time-dependent* observational data sets for data assimilation [34, 82]. The goal in these temporal inversions is to constrain model parameters based on the transient evolution of the system, rather than based on a snapshot or average. It requires a time-dependent adjoint model, typically derived by automatic differentiation [83, 84], which is applicable in principle where observations of sufficient spatial and temporal coverage are available. A working example of temporal inversion exists for the NEGIS [34], where SMB and basal friction are simultaneously optimised using observed changes in surface elevation. A similar method used to calibrate transient projections of a system of outlet glaciers based on surface elevation and velocity observations over a 10-year period has been applied in Antarctica [82]. This approach facilitates study of changes in inferred parameters in time as well as more accurate simulations of the evolution of glaciers’ dynamics. While these methods show great potential for large-scale application, their success will strongly depend on high quality observational data sets becoming available and the capability to scale this approach to large-scale systems.

Another opportunity to improve estimates of ice sheet configuration lies in the recent radar observation of ice isochrones [85] that have been reconstructed and dated over the entire GrIS [86]. These data sets contain invaluable information of the past evolution and conditions of the ice sheet and have the potential to revolutionise reconstructions of the ice sheet state if models are able to integrate all these data [83].

### Ice Sheet Model Validation

In order to validate models and assess their reliability, attempts have been made to compare present-day GrIS simulations to remote sensing observations. Comparison with Gravity Recovery and Climate Experiment (GRACE) data [87] for the short period of available observations starting in 2003 [20, 21, 88] highlighted good agreement between modelled and observed mass loss in regions dominated by SMB changes, and lower agreement in areas where dynamic changes are dominant. Given that the GRACE data set is growing and represents a largely independent source of information, complementing the data sets assimilated during initialisation, this exercise has the potential to develop into a powerful evaluation mechanism. Augmented by addition of a comparison with laser altimetry data [89], this procedure has been developed into a complete ice sheet model validation framework [88]. However, the applied ice flow models do not yet incorporate all the processes and forcing to simulate short-term dynamic changes arising from the variability of marine-terminating outlet glaciers and also lack representation of the long-term background evolution arising from past temperature changes [20, 21, 88], implying very large uncertainties. This limits any comparison focused on variability in SMB and ice flow response to recent SMB changes and allows the study of missing short- and long-term dynamical processes only on the basis of residual arguments. The recent acquisition of ice velocity with a high temporal resolution by, e.g., Landsat will improve comparisons of ice dynamics with observations and lead to advances in the representation of physical processes currently lacking in ice flow models.

## Interaction with the Climate System

Coupling with the atmosphere in terms of prescribing SMB and temperature boundary conditions in an ice sheet model is common practice and is not further discussed here. Instead, we refer the reader to a review paper on SMB [90]. However, additional challenges come into play when ice sheet models are fully coupled with climate models. A comprehensive review of coupled ice sheet–climate modelling and its challenges dates back to 3 years ago [91], so we only recapitulate some important points here and review the latest developments since then [92–95]. Two-way coupling requires careful consideration of the exchange of quantities to ensure mass and energy conservation and consistency between the different components. For example, water in solid, liquid and gas form need to be exchanged between ice sheet, climate and ocean models. A tool that facilitates that process is a flexible mapping system [94, 96, 97]. The mapping from one model component to the other is complicated by the need to bridge large differences in the spatial resolution between typically relatively coarse resolution climate models and high-resolution ice sheet models [81]. Recent results with the EC-earth model coupled to an ice sheet model indicate a critical role for the simulation of the surface albedo [95]. Often, the coupling requires downscaling techniques to better represent the SMB boundary condition, critical to achieve a realistic simulation of the ice sheet [92, 93, 95]. One technique is based on energy balance calculations for different elevation classes [92, 93, 98, 99], which allows for correcting the Atmosphere-Ocean General Circulation Model (AOGCM) grid-based results by the elevation difference with the ice sheet grid. This approach exploits the strong relationship between surface elevation and temperature to improve the accuracy of the SMB calculated on the atmospheric grid by downscaling to the ice sheet surface elevation at much higher spatial resolution. Alternatively, local spatial gradients of the SMB can be calculated [100] to directly account for changes in surface elevation in between coupling intervals of AOGCM and ice sheet model or for mismatches in surface elevation. The feedback between the different components of an AOGCM and ice sheet model requires adapted initialisation techniques that take into account differences in the response time of the individual components [101]. Often it is necessary to perform model initialisation of the ice sheet component separately, before coupling with the climate model.

With ISMIP6 [13], fully coupled GCM-ISM modelling has become an integral part of CMIP. In this framework, ad hoc methods to improve the representation of climate boundary conditions (e.g. anomaly forcing or flux correction) are systematically avoided, which may lead to biases in the simulations but guarantees fully physical and clearly interpretable results [13]. This approach appears to be appreciated by most of the fully coupled ice sheet–climate modellers and is an important complement to other methods when attempting to represent the present-day ice sheet as accurately as possible. However, on short time scales, downscaling techniques are unavoidable because of the need of a high spatial resolution for mass balance modelling of the GrIS, which is not yet feasible within full AOGCMs.

## Paleo Ice Sheet Simulations

Reconstructing the past evolution of the GrIS may have important implications for our understanding of its present and future behaviour, may provide analogues to the present-day climate and is a challenging scientific endeavour in itself. This is usually done with models representing not only the ice flow itself but also other components of the Earth system (atmosphere, ocean, solid Earth), often in parameterised or simplified form. While the spin-up of paleo-class ice sheet models to incorporate the long-term thermal memory typically covers the last or several glacial–interglacial cycles [15, 16], studying individual periods of the past with a specific focus is its own field of research. From its first inception during the Miocene ~ 10 million years ago [102] to its retreat during the last deglaciation [103], the GrIS has responded to changing climatic boundary conditions. Of particular interest is the GrIS behaviour during past warm periods like the mid Pliocene [104, 105] and the Last Interglacial (LIG) [15, 106–109], which may bear some resemblance to the expected future warming under anthropogenic greenhouse gas forcing. Once (partially) removed, the re-inception and sustainability of the ice sheet in cooling climatic conditions [109, 110] is of interest in view of potentially similar behaviour during a long-term future decrease of greenhouse gases in the atmosphere.

Sensitivity experiments with different topographic reconstructions were performed to study the conditions during the first inception of the GrIS [102]. The study concludes that growth of the ice sheets is only possible with help of tectonic uplift that produces favourable conditions for ice formation in high-elevation regions. GrIS (re-)inception and sustainability during the Late Pliocene was studied with another ice sheet model forced with GCM climate data under various sensitivity experiments [110]. Albeit operating in the framework of steady-state simulations, the results appear to support the hypothesis that no abrupt inception occurred but rather a cumulative regrowth of the ice sheet during favourable (cold) orbital configurations over several glacial–interglacial cycles.

An intercomparison of ice sheet models forced with simulated climate conditions of the mid-Pliocene warm period [104] indicates strong dependency on the assumed climatic boundary conditions. In contrast, a relative good agreement was found between the participating ice sheet models, which points to a limited sensitivity to ice dynamics. A companion paper complements the work with a study of the influence of several climate models on the response of one particular ice sheet model [105], which underlines the earlier finding and highlights the large uncertainties in climate boundary conditions for this period.

Simulations of the GrIS evolution during the LIG were performed with an ice sheet model asynchronously two-way coupled to a Regional Climate Model [106] to represent the atmospheric boundary condition with high detail. The results indicate a dominance of SMB forcing and a contribution at the lower end of the reported range of the GrIS to the LIG sea-level high-stand [1], suggesting an important contribution of the Antarctic ice sheet to sea level during this period or a possible contribution from Earth dynamics [111]. The importance of insolation forcing for the ice sheet evolution during this period was evaluated and confirmed with a fully coupled ice sheet–climate model of intermediate complexity [107]. Large ensemble simulations were performed for the LIG period and the present day to introduce and evaluate a novel discharge parameterisation [15], which helps to improve the margin position of the ice sheet. For the same period, an Earth system model of intermediate complexity with two-way coupled ice sheet components was used to study the sea-level evolution and ice–climate interactions in one consistent modelling framework [109]. A one-way coupled version of the same model [108] was used to evaluate the impact of ice sheet freshwater fluxes on the climate evolution and suggested a limited influence of GrIS meltwater fluxes in the presence of other, much larger ice sheet changes over Eurasia and North America at the onset of the LIG.

The retreat history of the GrIS during the last deglaciation was reconstructed using an ice sheet model constrained with evidence of past margin positions and relative sea-level indicators [103, 112]. The reconstruction provides important constraints for model spin-up to the present day, when representing the correct loading history and isostatic response.

Given the long-term experiments needed in paleo simulations, ice sheet and climate models and their coupling, are typically treated with reduced resolution and/or reduced complexity for synchronously coupled experiments [107–109, 113]. Because of the large contrast in resolution between climate and ice sheet models, the coupling is therefore often done with anomaly methods and/or requires downscaling techniques [113]. However, a continuous increase in the computational resources available to modellers has led to fully coupled AOGCM-ice sheet model simulations slowly becoming feasible for paleo simulations of multi-millennial time scales [114], albeit still with simplified mass balance schemes. It is evident that limitations of GCMs in simulating SMB boundary conditions and in the coupling discussed above for future projections equally apply for paleo applications but may appear less important in the light of other uncertainties arising from, e.g. poorly constrained boundary conditions and forcing in the paleo realm.

## Conclusions

We have discussed the literature of recent studies covering overlapping approaches to numerical modelling of the GrIS from paleo applications to future projections. The last few years have seen an important improvement in simulating the present-day dynamic state of the ice sheet, needed as an initial condition for projections of future ice sheet changes. Advances in data assimilation techniques that leverage the growing number of satellite and airborne observations of the ice sheets are at the core of these improvements and have led to modelled ice sheets that reproduce the observed velocity field much better. This is indicative of a growing link between the ice sheet modelling and observational communities, which will also pave the way towards better evaluation and validation mechanisms, which are needed to attach meaningful uncertainty estimates to the projections. However, the relatively short time coverage of satellite data is so far one of the biggest limitations to this effort and further progress is fundamentally dependent on a continuation of high-quality ice sheet observations over time, in order to allow for data assimilation techniques that take advantage of the temporal evolution. A limitation of the current generation of data-assimilated ice sheet models used for short-term projections is the missing representation of long-term processes of the bed response, thermodynamics and a prognostic evolution of the basal conditions. This is complemented by process-based models of long-term ice sheet evolution that typically include these physically based descriptions but often at lower spatial resolution and that exhibit considerable mismatch to the observed geometry and velocity at the present day. Recent development from this side of the modelling spectrum is therefore achieved by a continuous increase in model resolution and simulated detail and by an increasing size of model ensembles used for sensitivity studies and exploring various types of uncertainty.

Two-way coupling between ice sheet models and AOGCMs has seen significant improvements and is on the way to parity in performance with standalone ice sheet models for the goal of performing future projections on multi-centennial time scales. Aside from the intricate complications that arise from coupling two models with dynamic feedbacks between them, one of the main challenges and limitations has long been to produce realistic enough SMB boundary conditions for the ice sheet. Recent development in dynamic downscaling methods has led to an important step forward in this regard.

Paleo studies of GrIS evolution have focused on modelling past warm periods that may help to understand ice sheet behaviour in a warmer than present climate. However, large uncertainties in the climate forcing for the LIG and the mid-Pliocene warm period limit the ability to constrain past ice sheet behaviour and consequently inferences for the long-term future of the ice sheet.

## Outlook

Large challenges remain concerning how to combine “the best of both worlds” from long-term spin-up and data assimilation techniques. The goal would be to produce modern ice sheet initial conditions that are close to observations and at the same time include long-term processes like bedrock adjustment and evolving thermodynamics that contain the (thermal) memory of past climate changes. This likely requires joint efforts from different corners of the ice sheet modelling community and integration of time series of observations. A big step in this direction would be to define physically based descriptions of basal processes that could replace the inversion for basal drag by deterministic modelling.

An emerging development that is expected soon is two-way coupling of GrIS models with regional climate models for future projections. Several groups are currently working on what represents an important step forward to explicitly simulate interactions between evolving ice sheet and climate. Direct coupling between ice sheet models and AOGCMs is underway and should over the next few years become available for multi-centennial future projections, on time-scales where ice-climate feedbacks play an important role.

The global ice sheet modelling community has grown significantly in the last several years, leading to new approaches and models with larger user bases. Tighter collaboration between groups across intra-community boundaries should be continued, exemplified by intercomparison exercises like ISMIP6 [13], MISOMIP1 [115] and others, that can lead to standards (e.g., common data formats, application programming interfaces, …) that facilitate further development and model/model-component comparison and validation.
